# Rapid Change From Spontaneous Twin Anemia Polycythemia Sequence to Twin-to-Twin Transfusion Syndrome

**DOI:** 10.7759/cureus.81608

**Published:** 2025-04-02

**Authors:** Kenji Horie, Katsusuke Ozawa, Takafumi Okusa, Rika Sugibayashi, Seiji Wada, Hironori Takahashi

**Affiliations:** 1 Department of Obstetrics and Gynecology, Jichi Medical University, Shimotsuke, JPN; 2 Center for Maternal-Fetal, Neonatal and Reproductive Medicine, National Center for Child Health and Development, Tokyo, JPN

**Keywords:** cesarean section, fetoscopic laser surgery, middle cerebral artery, peak systolic velocity, twin anemia polycythemia sequence, twin-to-twin transfusion syndrome

## Abstract

A new natural course of spontaneous twin anemia polycythemia sequence (TAPS) was clarified. A 29-year-old woman, who conceived monochorionic diamniotic twins, showed elevation of the peak systolic velocity of the middle cerebral artery (MCA-PSV) in one twin at 24+2 weeks. The other twin showed a decrease in MCA-PSV. The amniotic volume did not differ between the twins. We diagnosed them with TAPS. Four days later, maximum vertical pockets were 19 and 97 mm in the donor and recipient, respectively. We diagnosed them with twin-twin transfusion syndrome, Quintero stage 1. Fetoscopic laser photocoagulation was employed, and the post-operative course was uneventful. Emergent cesarean section was conducted at 33+0 weeks because of premature rupture of the membranes. Neither twin showed anemia nor polycythemia. Only one patient with spontaneous TAPS followed by twin-to-twin transfusion syndrome (TTTS) has been reported so far. To our knowledge, this is the first report of a pregnant woman with TAPS developing TTTS within a few days.

## Introduction

The twin anemia polycythemia sequence (TAPS) shows a difference in the hemoglobin level via placental small arteriovenous (AV) anastomoses without a difference in amniotic fluid in monochorionic diamniotic (MD) twins. Most TAPS occurs following fetoscopic laser photocoagulation (FLP) for twin-to-twin transfusion syndrome (TTTS). The Quintero staging is the most widely used classification of TTTS, ranging from stage 1 to 5 [1]. Stage 1 is the presence of oligohydramnios (maximum vertical pocket: MVP less than 20 mm) and polyhydramnios (MVP more than 80 mm). Stage 2 is composed of an invisible bladder in the donor twin. Stage 3 shows abnormal Doppler flow of the umbilical artery and/or ductus venosus in one or both twins. Stage 4 shows hydrops fetalis in one or both twins. Stage 5 shows intrauterine fetal death in one or both twins. The standard treatment is FLP in patients with TTTS stages 2-4 at less than 26 weeks of gestation. On the other hand, TAPS can occur spontaneously and account for 3-5% of cases in MD twins [2,3]. Elevation of the peak systolic velocity of the middle cerebral artery (MCA-PSV) is the most important marker to detect TAPS antenatally. Placentas in MD twins have AV, arterio-arterial (AA), and venovenous (VV) anastomoses. AV anastomoses are unidirectional, and they are a key for the development of not only TTTS but also TAPS. In addition, the size and number of AV anastomoses are important in their formation. Regarding TTTS, large- or medium-sized AV anastomoses are causative vessels. Contrarily, in spontaneous TAPS, small-sized AV anastomoses are causative vessels [4]. In addition, the number of AV anastomoses is generally small in TAPS [4]. Spontaneous TAPS and TTTS are not continuously seen in the same patient because the key vessels are different. Here, we describe a very rare case of spontaneous TAPS followed by TTTS. Notably, TAPS developed into TTTS in only a few days.

## Case presentation

**Figure 1:**
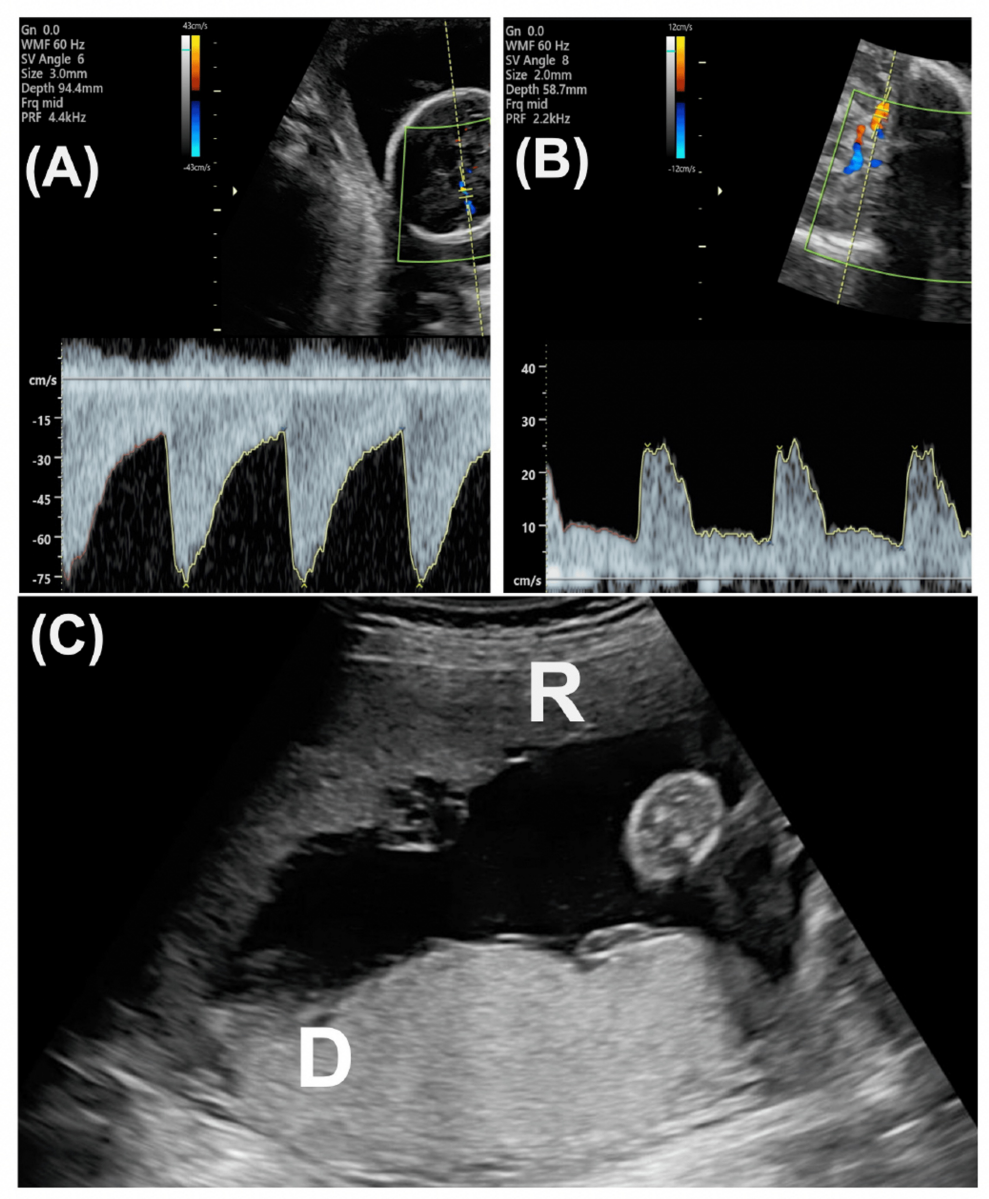
Fetal ultrasound at 24+5 weeks (A) Doppler flow of the middle cerebral artery (MCA) in the donor. The peak systolic velocity (PSV) was more than 75 cm/s. (B) Doppler flow of MCA in the recipient. MCA-PSV was less than 30 cm/s. (C) Placental findings on transabdominal ultrasound before the laser surgery. Gray-scale appearances are different between the donor and recipient. The placenta of the recipient (R) shows a black decoloration, and that of the donor (D) appears white and thick. R: recipient, D: donor

A 29-year-old (1-gravida, 0-parous) woman was referred to us at nine weeks of gestation for MD twin pregnancy. She conceived by frozen blastocyst transfer. No fetal structural abnormalities were noted, including nuchal translucency, in the first trimester. We observed her pregnancy course every two weeks. At 24+2 weeks, one fetus (donor) showed an elevated MCA-PSV, which was 82.3 cm/s (2.65 multiples of median: MoM), and the other fetus (recipient) showed decreased MCA-PSV, which was 20.29 cm/s (0.65 MoM). The estimated fetal body weight of the donor and recipient was 547 g (SD: -1.38) and 625 (SD: -0.63), respectively. The MVP was concordant (donor: 62 mm, recipient: 71 mm) in the two fetuses. No Doppler abnormalities were noted in the umbilical artery or ductus venosus of either fetus. We diagnosed the twin with TAPS. We cautiously observed the course twice weekly. At 24+5 weeks, MCA-PSV was 76.4 cm/s (2.41 MoM) in the donor and 24.2 cm/s (0.76 MoM) in the recipient (Figures 1A, 1B).

The amniotic discordance began to be seen (MVP: 44 mm in the donor, and 101 mm in the recipient). Regarding the placental findings, echogenicity of the placenta differed: donor’s and recipient’s placentas showed hyper-echogenicity and hypo-echogenicity, respectively (Figure 1C). We considered employing FLP and referred to the fetal treatment center. At 24+6 weeks, ultrasound showed that MVPs of the donor and recipient were 19 and 97 mm, respectively. The bladder was visible, with diastolic Doppler flow showing the umbilical artery in the donor. No abnormal Doppler flow pattern was noted in the recipient. We diagnosed her with TTTS stage 1. FLP to coagulate the vascular anastomoses on the placenta was employed by the Solomon method to improve the condition of TTTS at 25+0 weeks. Regarding anastomoses of the placenta, only a few small AV anastomoses were noted (Video 1).

**Video 1:** An operative video of the laser surgery Played in 3x speed

**Figure 2:**
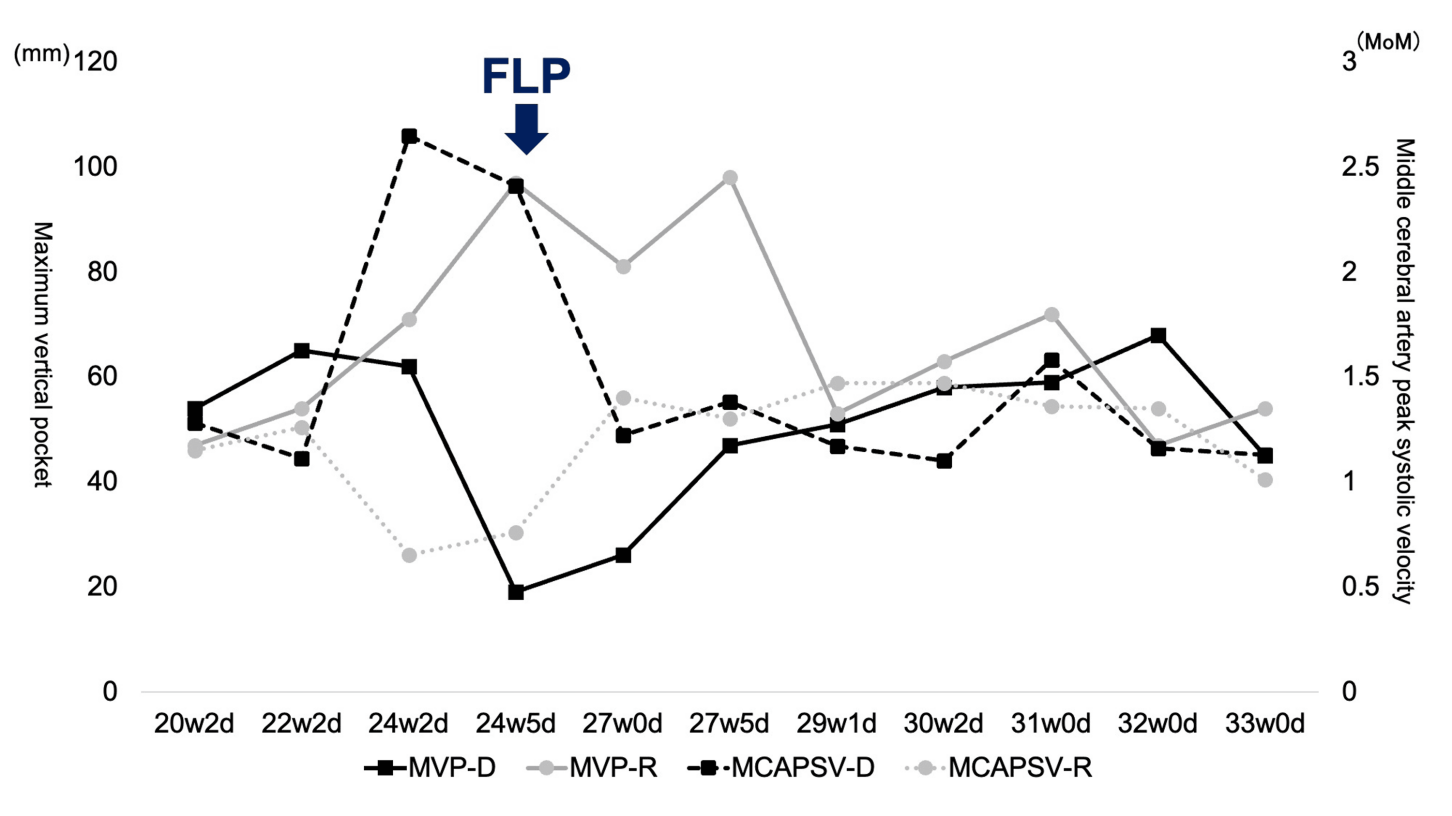
Prenatal course of the twin Each parameter markedly improved after the surgery. MVP-D: maximum vertical pocket of donor, MVP-R: maximum vertical pocket of recipient, FLP: fetoscopic laser photocoagulation

No AA or VV anastomoses were seen. The post-FLP course was uneventful (Figure 2). The MCA-PSV in both twins gradually returned to normal; thus, we considered that TAPS had also resolved.

The amniotic volume was also stable. We followed the course every week.

At 33+0 weeks, premature rupture of the membranes occurred. Tocolysis was induced by administering magnesium sulfate following betamethasone (12-mg intramuscular injection). At 33+1 weeks, emergent cesarean section was performed due to the onset of labor, yielding vigorous male infants (ex-donor: 1,729 g, Apgar score: 7/9 (1/5 min), umbilical artery pH: 7.301, hemoglobin: 15.6 g/dL; ex-recipient: 1,814 g, Apgar score: 8/9 (1/5 min), umbilical artery pH: 7.298, hemoglobin: 16.3 g/dL). The neonates were admitted to the neonatal intensive care unit. Both were temporarily intubated. Neither intraventricular nor gastrointestinal hemorrhage was observed. Brain MRIs were also normal. They were discharged without sequelae at 40 days.

**Figure 3:**
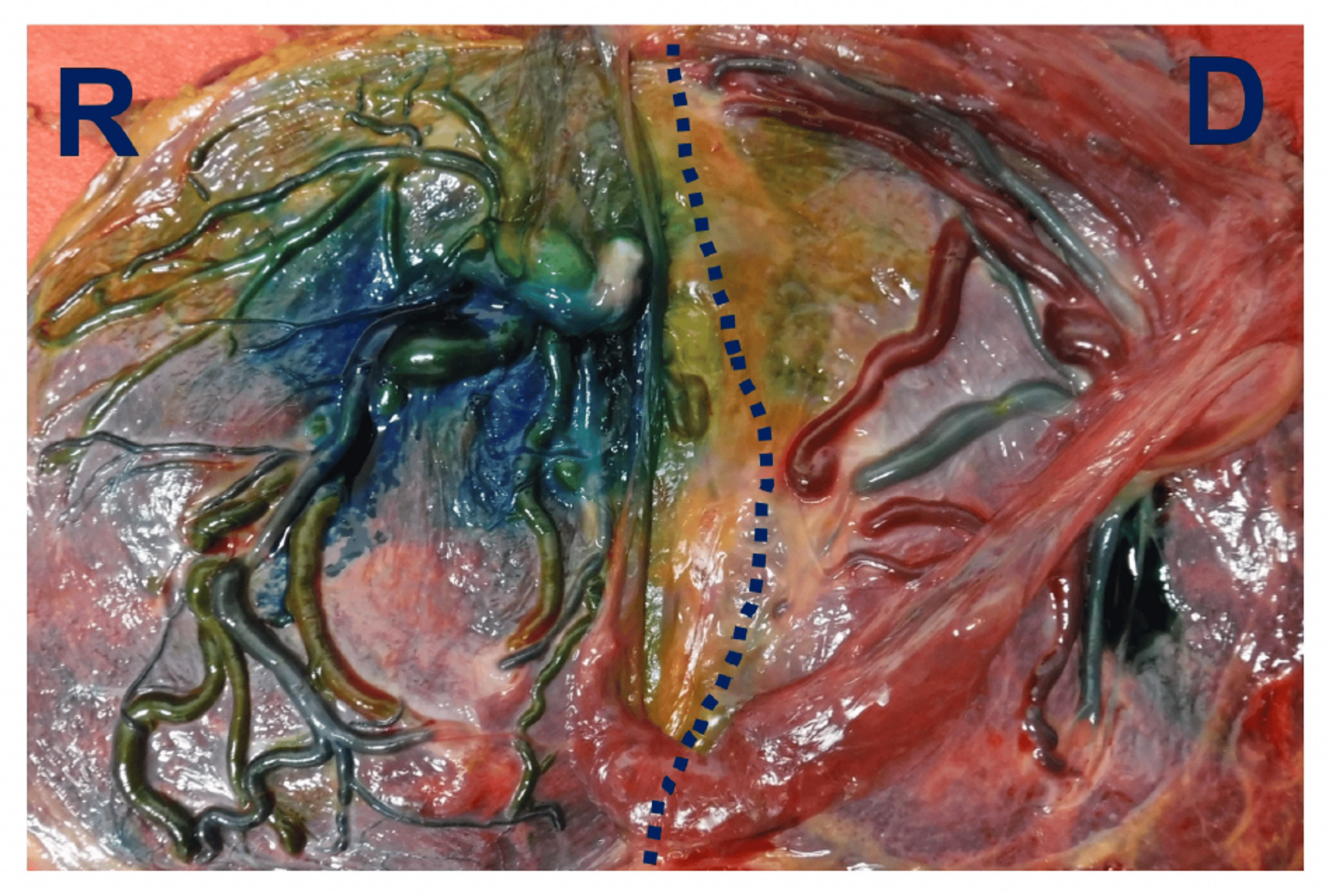
Fetal side of the placenta following dye injection Yellow and blue vessels show veins and arteries of the recipient, respectively. Red and green vessels show veins and arteries of the donor, respectively. The dotted line is the coagulation line. Regarding shunt vessels, only a few small arteriovenous anastomoses existed around the dotted line. Small amount of stain leaked out. D: donor side, R: recipient side

Regarding placenta findings, all AV anastomoses were coagulated (Figure 3).

There were only a few small AV anastomoses, which were coagulated completely. No AA or VV anastomosis was noted. Additionally, no chorioamnionitis or retroplacental hematoma was observed.

## Discussion

Based on our PubMed search, this is the first report of a pregnant woman with TAPS developing into TTTS within a few days. A new natural course of spontaneous TAPS has been clarified.

It is interesting to note that TTTS developed from TAPS within a few days. Only one patient in whom spontaneous TAPS was followed by TTTS has been reported [5]. In that case [5], TAPS manifested at 20 weeks and gradually worsened until 22 weeks. Then, amniotic discordance appeared, resulting in TTTS stage 1 at 24 weeks. FLP was performed at 24+6 weeks. It took approximately four weeks to develop TTTS from TAPS in the reported case [5]. In contrast, the amniotic fluid volume changed rapidly in our case. At 24+2 weeks, TAPS was diagnosed, and amniotic discordance was not noted (MVP: 62 mm in the donor and 71 mm in the recipient). However, three days later (24+5 weeks), amniotic discordance began to be observed, and TTTS stage 1 was diagnosed the following day. It took only four days.

Although we do not know why the marked change occurred in our case, anastomosis vessels may be key to the rapid change. Interestingly, only a few small AV anastomoses were observed in our case. Monochorionic placentas generally contain AV, AA, and VV anastomoses. AV anastomoses are usually unidirectional, whereas both AA and VV anastomoses are bidirectional. Among these vascular anastomoses, small AV anastomoses cause slow red blood cell shift and contribute to the development of TAPS. The median number of AV anastomoses in normal monochorionic twins, selective fetal growth restriction, and TTTS placentas was 7 (interquartile range (IQR): 3-10), 6 (IQR: 4-13), and 6 (IQR: 4-10), respectively. The number was significantly less in the TAPS placenta (median 4 (IQR: 2-5); p < 0.01). Furthermore, the median diameter of AV anastomoses in TAPS was significantly smaller compared with that in TTTS (TAPS: 0.1 mm (IQR: 0.1-0.2) vs. TTTS: 0.4 mm (IQR: 0.3-0.6); p<0.01) [6]. Our case and the reported case [4] in which TAPS was followed by TTTS had different characteristics of anastomoses. In the reported case, one large AV, three medium‐sized AV, and three small unknown anastomoses were detected [5]. In our case, only a few small AV anastomoses were observed, and middle or large anastomoses were not detected. Some of those small anastomoses may surely be associated with the rapid development of TTTS, although the trigger of a rapid blood shift was unknown. AA anastomoses are seen in 10-20% of TAPS placentas and are smaller in placentas with TAPS than in placentas of uncomplicated MD twins [4]. We could not detect AA anastomoses at surgery and post-laser placental histology. The bidirectional blood flow at normal AA anastomoses usually shows a protective effect for the development of TTTS [7]. The placenta in our case did not have AA anastomoses; thus, an unidentified trigger easily induced the abrupt amniotic fluid volume imbalance, resulting in TTTS.

TAPS developed spontaneously or resulted from post-laser surgery. According to a registry from 17 perinatal centers, of 366 TAPS cases, 216 (59%: 216/366) were spontaneous [8]. Spontaneous TAPS was detected at a median of 23.7 (IQR: 19.7-28.8) weeks [9]. In our case, we noticed TAPS at 19 weeks. Occurrence at less than 20 weeks accounts for only 25% of all cases.

There is no established treatment for TAPS. Various forms of antenatal management, including FLP, expectant management, delivery, intrauterine transfusion, selective feticide, and termination of pregnancy, were attempted. The choice of treatments may depend on the severity of TAPS and gestational weeks. At 32 weeks or more, an emergent cesarean section is recommended. Before then, for stages 1 and 2 (MCA-PSV difference only), course observation may be selected. We need to consider that TAPS resolved in 16% of patients [8]. Stage 3 or more (MCA-PSV difference plus abnormal Doppler or hydrops fetalis) requires interventions. The coagulation of AV anastomoses is the best option for disease improvement before 28 weeks of gestation. However, coagulation for TAPS is technically difficult. The scope is inserted into the cavity of polyhydramnios in TTTS. However, it is difficult to insert the scope in a case of TAPS because of the narrow cavity. To overcome the problem, amnioinfusion or amnioreduction is employed in surgery. In our case, we planned to perform amnioinfusion for TAPS treatment before FLP. If difficult to perform, we considered intraperitoneal transfusion for the donor.

The outcome of spontaneous TAPS is not good. Indeed, severe neonatal morbidity occurred in 33% (141/432) of TAPS cases. Donors (32%: 63/196) and recipients (33%: 75/228) had similar severe neonatal morbidity [8]. Long-term outcomes of spontaneous TAPS in 49 cases were unfavorable [7]. Neurological impairments were observed in 30% of survivors, and donors are more severely affected (donors 44% vs. recipients 18%).

## Conclusions

TAPS may be a high-risk condition that progresses to TTTS. Regular ultrasound check-ups, including MCA-PSV measurements, are essential for monitoring monochorionic diamniotic twins. If MCA-PSV discordance is observed, obstetricians should pay attention to not only TAPS but also the development of TTTS. On the other hand, when patients with MD twins show amniotic imbalance, the occurrence of TAPS may be considered.
